# PET/MRI Applications in Pediatric Epilepsy

**DOI:** 10.1055/s-0043-1764303

**Published:** 2023-04-28

**Authors:** Christian Pedersen, Mariam Aboian, Steven A. Messina, Heike Daldrup-Link, Ana M. Franceschi

**Affiliations:** 1 Department of Radiology, Yale School of Medicine, New Haven, Connecticut, United States; 2 Neuroradiology Division, Department of Radiology, Mayo Clinic Radiology, Rochester, Minnesota, United States; 3 Department of Radiology and Pediatrics, Stanford University School of Medicine, Palo Alto, California, United States; 4 Neuroradiology Division, Department of Radiology, Northwell Health/Donald and Barbara Zucker School of Medicine, Lenox Hill Hospital, New York, New York, United States

**Keywords:** hybrid imaging, PET/MRI, pediatric epilepsy, malformations of cortical development, focal cortical dysplasia, temporal lobe epilepsy, mesial temporal sclerosis, tuberous sclerosis

## Abstract

Epilepsy neuroimaging assessment requires exceptional anatomic detail, physiologic and metabolic information. Magnetic resonance (MR) protocols are often time-consuming necessitating sedation and positron emission tomography (PET)/computed tomography (CT) comes with a significant radiation dose. Hybrid PET/MRI protocols allow for exquisite assessment of brain anatomy and structural abnormalities, in addition to metabolic information in a single, convenient imaging session, which limits radiation dose, sedation time, and sedation events. Brain PET/MRI has proven especially useful for accurate localization of epileptogenic zones in pediatric seizure cases, providing critical additional information and guiding surgical decision making in medically refractory cases. Accurate localization of seizure focus is necessary to limit the extent of the surgical resection, preserve healthy brain tissue, and achieve seizure control. This review provides a systematic overview with illustrative examples demonstrating the applications and diagnostic utility of PET/MRI in pediatric epilepsy.

## Introduction

Epilepsy is a common chronic neurologic condition in childhood, affecting 0.5 to 1% of children. 1 Drug-resistant epilepsy occur in approximately 25% of pediatric patients. 2 3 Meanwhile, surgical treatment is effective but invasive and requires careful preoperative planning. 4 5 6 A multidisciplinary and multimodal imaging approach with functional PET and anatomical MRI components is necessary to accurately localize epileptogenic foci and limit extent of surgical resection and thus postoperative physical and neuropsychological deficits. 7 A diagnostic algorithm for pediatric epilepsy should ideally include semiology, interictal and ictal positron emission tomography (PET), and invasive monitoring such as stereoelectroencephalography (SEEG) or placement of surface subdural grids to guide a targeted anatomic and metabolic review for overlooked subtle abnormalities. Although invasive, SEEG is considered the gold standard for spatiotemporal definition of seizure-onset zone in the ictal period.

**Fig. 1:**
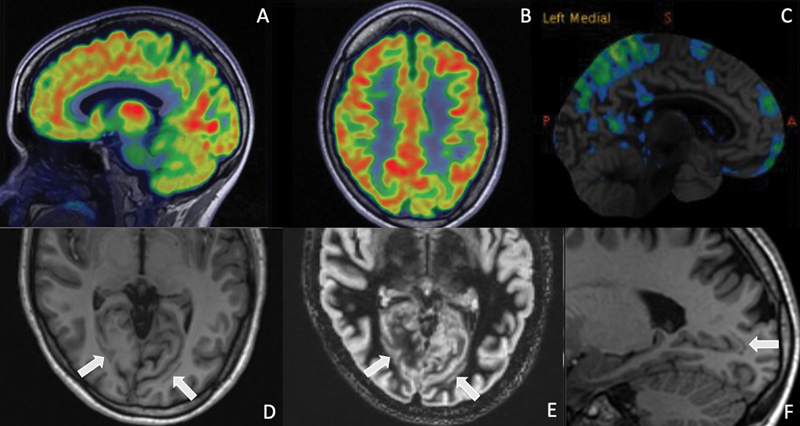
^18^ F-FDG-PET ( **A, B** ) and automated voxel by voxel z-score database map (Cortex ID, GE Healthcare) ( **C** ) shows focal areas of hypometabolism in the parietal and occipital lobes, more pronounced on the left side, which resulted in focused review of corresponding MRI. Focal hypometabolism corresponds to gray matter bands adjacent to the occipital horns of the lateral ventricles on the MPRAGE and DIR sequences ( **D–F** ), which were isointense to cortex on all sequences, and compatible with band heterotopia.

^18^ F-fluorodeoxyglucose (FDG) is the most common PET radiotracer used in clinical assessment of epilepsy patients, and in one survey, ^18^ F-FDG-PET was shown to provide critical additional information on the localization of epileptogenic zone in 77% of cases and guide surgical decision making in 51% of cases. 8 Epileptogenic zones are characterized by focal hypometabolism on ^18^ F-FDG brain PET. Hypometabolism associated with an underlying structural abnormality, such as malformations of cortical development (MCD), may be greater than the size of the lesion identified by structural imaging and the epileptogenic zone defined by invasive monitoring. 9 10 For example, Fig. 1 demonstrates a rare form of band heterotopia, 11 which was challenging to detect on magnetic resonance imaging (MRI) but much more conspicuous on PET imaging. Interictal ^18^ F-FDG-PET is more sensitive than MRI in localizing epileptogenic seizure nidus in both temporal lobe and extra-temporal epilepsy and may successfully identify MCD despite negative MRI. 12 13 Notably, a recent study investigated regional metabolic rate of glucose (MRGlc) derived from the analysis of FDG PET and MRI data. The authors found that MRGlc abnormality maps contributed to the localization of hypo-metabolic areas in 53% of cases when comparing patients with non-lesional extratemporal epilepsy to a normative database, but the overall quantitative PET imaging was only found to have a moderate impact on clinical management. 14

More recently, epileptogenic alterations in the central diazepine receptor system have been used in the localization of epileptogenic foci by utilizing the ^11^ C-flumazenil (FMZ) radiotracer, which has high affinity for GABA _A_ receptors. ^11^ C-FMZ PET demonstrates interictal decreased binding in the seizure-onset zone and may provide valuable information to complement MRI and FDG-PET in presurgical evaluation. 15 The major limitation is the very short half-life of ^11^ C (approximately 20 minutes), which limits the use primarily to the research setting and institutions with an on-site cyclotron. ^11^ C-alpha-methyl-L-tryptophan (AMT) is a tryptophan analog that traces serotonin metabolism and demonstrates increased interictal activity in the epileptogenic cortex. ^11^ C-AMT PET has shown promise in patients with tuberous sclerosis and MCD due to specificity approaching 100% and smaller area of abnormality compared to FDG or FMZ PET. 16 AMT-PET does not require EEG monitoring and performs well even in the setting of partial volume artifacts caused by focal atrophy in the epileptogenic region. Similar to FMZ PET, it currently has limited clinical utility.

Neuroinflammation has also been implicated in epileptogenesis. PET radioligands include translocator protein (TSPO) tracers. TSPO is an 18-kDa protein found on the outer mitochondrial membrane of activated microglia and astrocytes, and therefore considered a tissue-specific biomarker of inflammation. The majority of the data supporting TSPO as a potential target for anti-inflammatory treatment in epilepsy are either preclinical or from limited clinical studies, and it is still not clear if TSPO is a more composite measurement of neuroinflammation or if it is specific enough to warrant targeted therapy. 17

Perfusion imaging for epilepsy using ^15^ O-H _2_ O PET has not gained widespread popularity given discordance and low diagnostic accuracy between perfusion and metabolic abnormalities in epileptogenic zones. 18 Various additional radioligands are also being evaluated in epilepsy, including ligands targeted for opioid receptors, histamine receptors, N-methyl-D-aspartate (NMDA) receptors, and acetylcholine receptors, aiming to provide more specific imaging guidance to improve results following surgical intervention.

Brain PET traditionally uses CT for attenuation correction and anatomic correlation. The inherent tissue contrast resolution associated with CT is suboptimal for the evaluation of structural abnormalities related to epilepsy; therefore, necessitating a separately performed brain MRI. Hybrid PET/MRI has become increasingly more common and enables improved accuracy of brain PET and MR image fusion, however, not without some pitfalls. Attenuation correction of brain PET data from PET/MRI uses advanced image-processing techniques and is the most commonly performed with an atlas-based approach, relying on a previously compiled atlas of paired MRI and CT exams to perform a pseudo-CT from the patient's MRI for subsequent attenuation correction, as opposed to utilizing direct information from the patient. Direct attenuation correction can also be performed with MRI using dixon, ultra-short echo, or zero-echo time sequences. In a cohort of children with focal epilepsy, the diagnostic accuracy of PET/MRI was not inferior to PET/CT for seizure focus localization. 19 A more recent study comparing PET/MRI with PET/CT and standalone MRI showed that diagnostic accuracy and image quality as determined by visual score were the highest in PET/MRI. 20

Hybrid PET/MRI can be obtained with synchronous or sequential systems. A sequential system is based upon patient transportation between PET and MRI scans, which are performed separately. The sequential system is clinically easier to implement because most hospitals own separate PET and MRI scanners. A major drawback is the asynchronous acquisition of the scans, which results in a longer scan and sedation time and misregistration artifacts. A synchronous system is technically more difficult to achieve but preferable in pediatric patients 21 because the solid-state PET detectors are located between the MRI body and gradient coils in one integrated machine, which allows for truly synchronous image acquisition with reduced scan and sedation time, and reduced risk of misregistration artifact. 22 23 Synchronous acquisition also ensures that both scans are obtained at the same time with regard to seizure occurrence given that dis-synchronous assessment may be misleading if scans are obtained distant in time from each other. As outlined in the 2019 consensus report from the International League Against Epilepsy Neuroimaging Task Force, 24 epilepsy protocols benefit from the inclusion of 3D volumetric T1-weighted imaging, axial and coronal T2-weighted images FSE or TSE, axial and coronal FLAIR sequences, and axial T2* gradient echo or SWI sequences. Oblique coronal T2-weighted images of the hippocampus, DWI/DTI, and ADC maps may also prove valuable. Gadolinium-enhanced images are not routinely indicated and reserved for cases with suspected infection, vascular abnormality, or tumors. Furthermore, higher field strength magnets such as 3- or 7-Tesla (T), provide better lesion detection and contrast resolution compared to 1.5 T magnets. For example, in one study, 15 out of 23 patients with medically refractory epilepsy and a normal 1.5 T brain MRI interpreted at a tertiary care center, had abnormal findings on 3T imaging. 25

Additional advanced neuroimaging modalities such as functional MRI (fMRI), single-photon emission computerized tomography (SPECT), magnetic resonance spectroscopy (MRS), and magnetoencephalography (MEG) may also be utilized in preoperative planning. 26 27 28 29 30 31 32 33 Functional MRI with simultaneous EEG monitoring can be helpful for the localization of epileptogenic foci as interictal epileptic discharges may show corresponding BOLD response on fMRI. Resting state functional MRI (rs-fMRI) has shown promise 26 in determining seizure-onset localization in nearly 90% of cases and altered surgery in almost 60% of cases in one single institution series. 27 Fiber-tracking techniques can advantageously be obtained for temporal lobe epilepsy, which allows the evaluation of reorganization of linguistic and associated functions. 28 29 Diffusion tensor imaging (DTI) can be used for preoperative evaluation of lateralization of motor cortex and delineation of corticospinal tracts, and has been shown helpful to predict postoperative motor outcomes. 30 DTI may also prove helpful in patients who fail to obtain seizure freedom after hemispherectomy by identifying transcallosal fibers responsible for residual interhemispheric connections. These patients may benefit from additional surgery. 31 Similar to FDG-PET, SPECT allows quantitative and qualitative assessments of cerebral blood flow, which is regionally increased in the ictal state and decreased interictally. A combination of interictal and ictal SPECT by statistical parametric mapping has been shown to help localize seizure foci, particularly in temporal lobe epilepsy. 32 In subtraction SPECT, the interictal SPECT is subtracted from the ictal SPECT and then co-registered with MRI to increase the accuracy of SPECT. 33 Challenges in SPECT imaging include identification of clinical onset of seizures for ictal imaging and larger anatomic areas of hyper perfusion compared to MRI and EEG findings.

### Focal Cortical Dysplasia

**Fig. 2:**
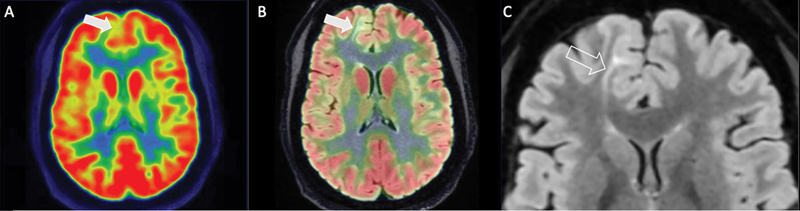
Focal hypometabolism along the medial right frontal lobe ( **A** ) corresponds to underlying cortical and subcortical irregularity on fused FLAIR PET/MRI sequence ( **B** ) with gray–white junction blurring, hyperintense T2 signal and positive transmantle sign, best defined on FLAIR sequence ( **C** ).

**Fig. 3:**
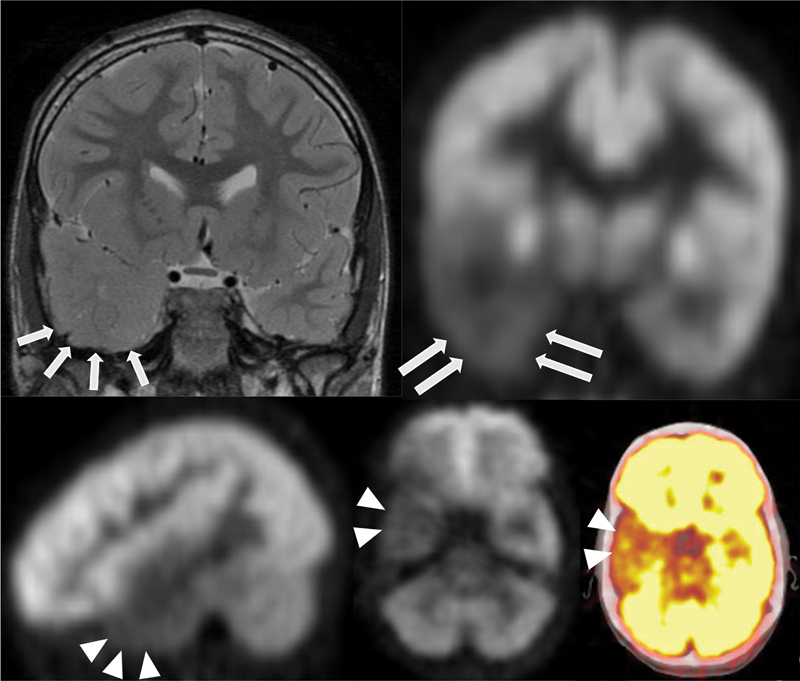
Brain MRI in patient with focal cortical dysplasia visualized as abnormal cortical thickening and effacement of white matter in the right temporal pole, best visualized on thin coronal T2-weighted MRI ( *arrows* , **A** ). On ^18^ F-FDG-PET ( *arrows* and *arrowheads* , **B–D** ) and fused axial ^18^ F-FDG PET/CT ( *arrowheads* , **E** ), there is corresponding hypometabolism in the right anterior temporal lobe.

Focal cortical dysplasia (FCD) is the most common MCD in pediatric medically refractory epilepsy and the most frequent histopathologic diagnosis in children undergoing epilepsy surgery. 34 35 FCD may present with blurring of the gray–white matter junction ( Fig. 2 ), cortical thickening ( Fig. 3 ), hyperintense T2/FLAIR signal of gray or white matter, transmantle sign ( Fig. 2 ), segmental or lobar atrophy or hypoplasia, and abnormal sulcal or gyral pattern. MRI is the main diagnostic modality in evaluation although some cases reveal very little to no apparent abnormality. In a study of 48 children, ^18^ F-FDG-PET demonstrated an advantage over MRI alone, with a sensitivity of 87% for FDG-PET versus 77% for MRI alone. 36 With hybrid imaging, the sensitivity of ^18^ F-FDG-PET can be increased by co-registration of color-coded PET images with MRI, which improves the detection of morphologic abnormalities such as FCD type I, with up to 82% of patients seizure-free after resection guided by PET/MR findings and electrocorticography. 13 36 Notably, ^18^ F-FDG-PET detects areas of hypometabolism in 60 to 92% of epilepsy patients with FCD. 37

In the absence of a focal lesion on MRI and surface EEG without localizing information, children undergoing presurgical evaluation may undergo ^18^ F-FDG-PET to obtain lateralizing and localizing information regarding epileptogenic cortex. 38 39 Identification of an epileptogenic zone and underlying lesion allows for surgical resection and increases the probability of seizure freedom. 40 Localized ^18^ F-FDG-PET abnormalities were reported in 84% of cases in a surgical series with patients with temporal lobe epilepsy and negative MRI. 41

### Pediatric Temporal Lobe Epilepsy

**Fig. 4:**
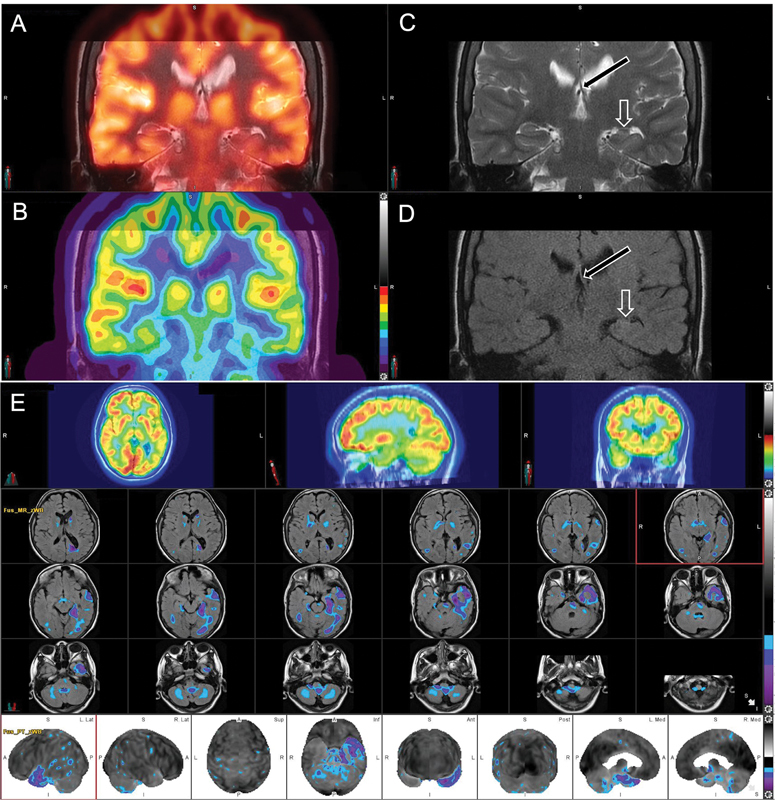
A 11-year-old male patient with medically refractory epilepsy. EEG demonstrated left temporal lobe seizure focus. Fused ^18^ F-FDG PET/MRI ( **A, B** ), coronal T2 and FLAIR ( **C, D** ) views demonstrate focal hypometabolism in the left temporal pole involving the left hippocampal formation, entorhinal cortex, and amygdala. There is decreased volume of the left hippocampal formation ( *open arrow* ) with abnormal morphology and subtle T2/FLAIR hyperintensity, and with associated decreased caliber of the left fornix ( *black arrow* ) and smaller size of the left mamillary body (not shown). Semiquantitative analysis using Z scores calculated in comparison to age-matched normal controls ( **E** ) reveals significantly decreased values in the left temporal lobe including in the left temporal pole, hippocampus, parahippocampal gyrus, and amygdala.

Temporal lobe epilepsy (TLE) is the most common medically intractable epilepsy in adults, with mesial temporal sclerosis (MTS) as the underlying cause in approximately 60% of cases. Tumor, MCD, trauma, nonspecific gliosis, and vascular malformations are less frequent etiologies. 6 42 43 Contrarily, only about 20% of pediatric patients have TLE, and only 20% of these children have MTS ( Fig. 4 ). 44 In the adult literature, there has been recognition of MRI-negative, ^18^ F-FDG-PET-positive TLE, with surgical seizure outcomes comparable to MRI-positive patients with mesial temporal sclerosis. 43 45

Reports demonstrate MCD as a common underlying histopathology in pediatric patients with pure temporal lobe epilepsy, with a predominance of type 1B and 2A focal cortical dysplasia. 46 It is important to keep in mind that many patients with pediatric TLE actually have temporal plus nosology. While detection of a lesion on MRI remains an important preoperative determinant of long-term seizure freedom following pediatric temporal lobectomy, ^18^ F-FDG-PET has proven invaluable in the workup of pediatric patients with refractory epilepsy.

### Tuberous Sclerosis

**Fig. 5:**
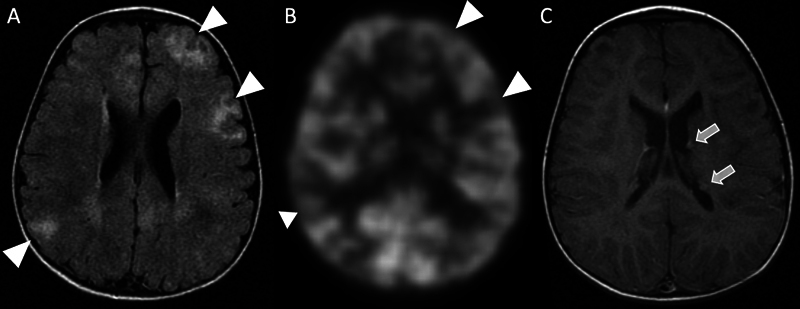
Brain MRI in a patient with tuberous sclerosis demonstrates multiple FLAIR hyperintense cortical tubers ( *arrowheads* , **A** ). The regions of the tubers appear to be hypometabolic on corresponding ^18^ F-FDG-PET ( *arrowheads* , **B** ). There are small enhancing subependymal nodules ( *arrows* ) along the left lateral ventricle, which are consistent with diagnosis of TS ( **C** ).

Epilepsy develops in 80 to 90% of tuberous sclerosis patients, in some as early as infancy, and approximately 25 to 50% of TSC patients may develop medically intractable epilepsy. 44 47 Intractable seizures can have a detrimental effect on neurocognitive development and early epilepsy surgery may be beneficial. 48 Cortical tubers are typically seen as multifocal areas of glucose hypometabolism, which is hypothesized to be due to decreased number of neurons and simplified dendritic pattern within the tubers with less requirement for glucose. The area of hypometabolism is often larger than the lesions seen on MRI. 49 50 Structural imaging typically demonstrates multiple bilateral subcortical tubers, but seizures often arise primarily from a single tuber. 51 Multimodality imaging algorithms utilizing MRI and ^18^ F-FDG-PET co-registration can play a valuable role by noninvasively localizing epileptogenic tubers during pre-surgical evaluation. 52 Notably, AMT-PET is unique because it can differentiate epileptogenic from non-epileptogenic tubers, which is helpful to guide surgery. 15 49 Surgical outcome studies demonstrate decrease in seizure burden after resection of the suspected epileptogenic tubers while leaving the non-epileptogenic tubers untouched. 53 54 Fig. 5 shows a case of tuberous sclerosis with multiple hypometabolic cortical tubers, which was managed non-surgically.

### Hemispheric Abnormalities

**Fig. 6:**
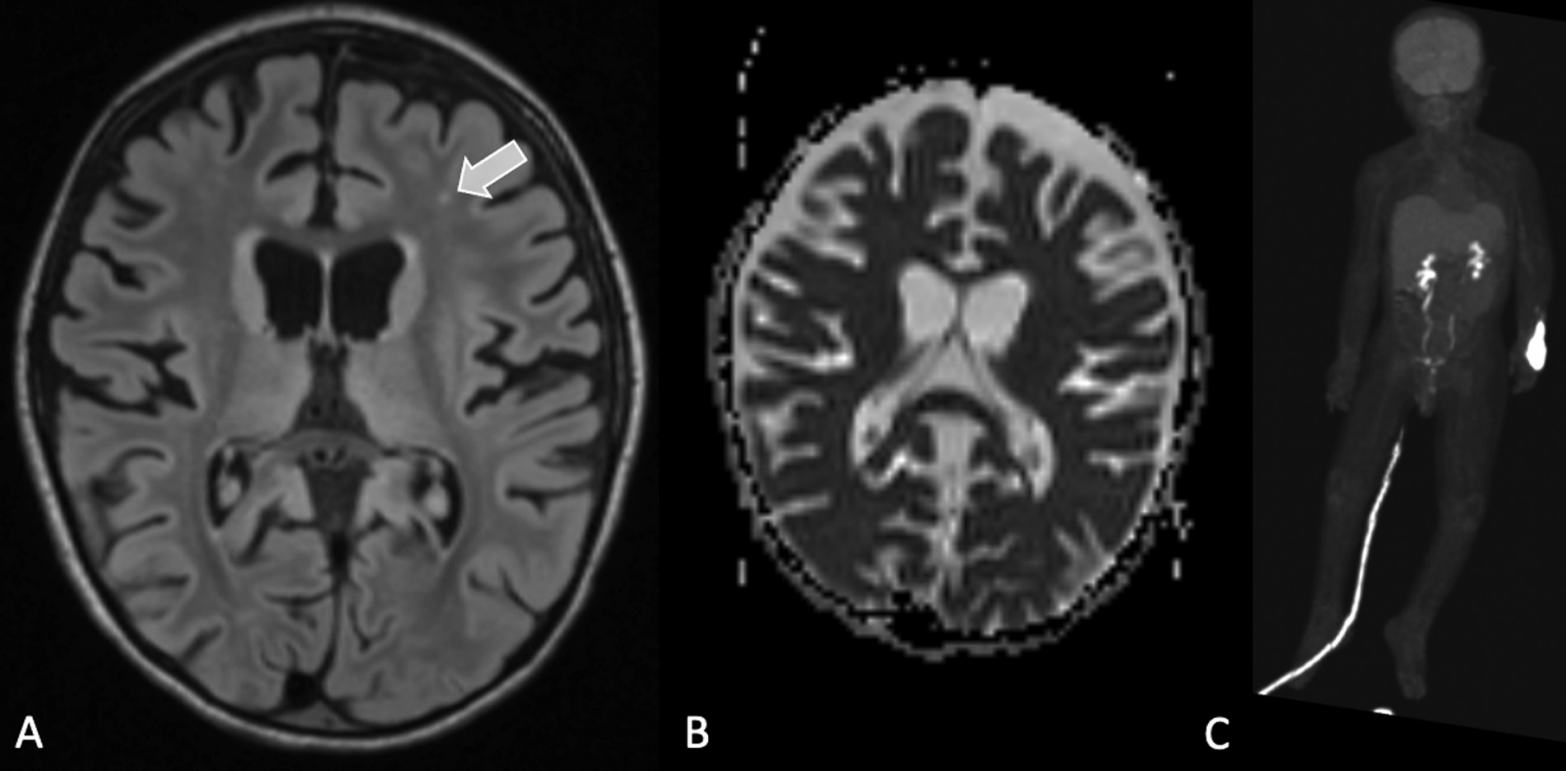
A 3-year-old child with refractory status epilepticus. MRI brain demonstrates parenchymal volume loss and white matter FLAIR hyperintense foci ( **A, B** ). Extensive laboratory testing was negative for arbovirus, AQP4 antibody, myelin oligodendrocyte glycoprotein, and SARS CoV-2. N-methyl-D-Aspartate IgG antibodies were detected. Subsequent ^18^ F-FDG-PET was performed to identify primary malignancy, which was negative ( **C** ).

^18^ F-FDG-PET is beneficial in evaluating patients with hemispheric abnormalities, including encephalomalacia, Rasmussen's encephalitis, and diffuse MCD. 55 These patients may develop intractable epilepsy and undergo evaluation for hemispherectomy, which is an effective surgical approach in children with multilobar or hemispheric epileptogenic lesions. In these cases, published large-sample studies report a rate of seizure remission of 65 to 80%. 56 ^18^ F-FDG-PET often demonstrates diffuse hypometabolism affecting the involved hemisphere. However, patients with bilateral ^18^ F-FDG-PET abnormalities have unfavorable seizure outcomes following hemispherectomy, emphasizing the benefits of ^18^ F-FDG-PET in pre-surgical assessment. 57 Fig. 6 shows a case of N-methyl-D-aspartate (NMDA) encephalitis, which may be associated with extra-cerebral malignancy. 58

## Conclusion

Diagnosis and treatment of pediatric epilepsy are complicated and requires a multidisciplinary and multimodal imaging approach. Accurate localization of a seizure focus is paramount for optimal treatment and favorable outcomes following surgical resection. Hybrid brain ^18^ F-FDG PET/MRI is an optimized imaging approach that allows for comprehensive multimodal imaging evaluation with reduction in radiation dose, sedation events, and sedation time.
